# The Key Driver Implementation Scale (KDIS) for practice facilitators: Psychometric testing in the “Southeastern collaboration to improve blood pressure control” trial

**DOI:** 10.1371/journal.pone.0272816

**Published:** 2022-08-24

**Authors:** Angela M. Stover, Mian Wang, Christopher M. Shea, Erica Richman, Jennifer Rees, Andrea L. Cherrington, Doyle M. Cummings, Liza Nicholson, Shannon Peaden, Macie Craft, Monique Mackey, Monika M. Safford, Jacqueline R. Halladay

**Affiliations:** 1 Department of Health Policy and Management, University of North Carolina at Chapel Hill, Chapel Hill, NC, United States of America; 2 Lineberger Comprehensive Cancer Center, Chapel Hill, NC, United States of America; 3 Cecil G. Sheps Center for Health Services Research, University of North Carolina at Chapel Hill, Chapel Hill, NC, United States of America; 4 NC Tracs Institute, University of North Carolina at Chapel Hill, Chapel Hill, NC, United States of America; 5 University of Alabama Birmingham, School of Medicine, Birmingham, AL, United States of America; 6 East Carolina University, Greenville, NC, United States of America; 7 Department of Public Health, Samford University, Birmingham, AL, United States of America; 8 Area L Area Health Education Center (AHEC)—Part of the NC AHEC Program, Rocky Mount, NC, United States of America; 9 Weill Cornell Medicine, New York, NY, United States of America; 10 School of Medicine, University of North Carolina at Chapel Hill, Chapel Hill, NC, United States of America; Prince Sattam Bin Abdulaziz University, College of Applied Medical Sciences, SAUDI ARABIA

## Abstract

**Background:**

Practice facilitators (PFs) provide tailored support to primary care practices to improve the quality of care delivery. Often used by PFs, the “Key Driver Implementation Scale” (KDIS) measures the degree to which a practice implements quality improvement activities from the Chronic Care Model, but the scale’s psychometric properties have not been investigated. We examined construct validity, reliability, floor and ceiling effects, and a longitudinal trend test of the KDIS items in the Southeastern Collaboration to Improve Blood Pressure Control trial.

**Methods:**

The KDIS items assess a practice’s progress toward implementing: a clinical information system (using their own data to drive change); standardized care processes; optimized team care; patient self-management support; and leadership support. We assessed construct validity and estimated reliability with a multilevel confirmatory factor analysis (CFA). A trend test examined whether the KDIS items increased over time and estimated the expected number of months needed to move a practice to the highest response options.

**Results:**

PFs completed monthly KDIS ratings over 12 months for 32 primary care practices, yielding a total of 384 observations. Data was fitted to a unidimensional CFA model; however, parameter fit was modest and could be improved. Reliability was 0.70. Practices started scoring at the highest levels beginning in month 5, indicating low variability. The KDIS items did show an upward trend over 12 months (all p < .001), indicating that practices were increasingly implementing key activities. The expected time to move a practice to the highest response category was 9.1 months for standardized care processes, 10.2 for clinical information system, 12.6 for self-management support, 13.1 for leadership, and 14.3 months for optimized team care.

**Conclusions:**

The KDIS items showed acceptable reliability, but work is needed in larger sample sizes to determine if two or more groups of implementation activities are being measured rather than one.

## Introduction

Practice facilitation is an evidence-based method for integrating research evidence into routine care delivery [1–3]. Practice facilitators (PFs), sometimes called “practice coaches,” are the agents providing tailored support on how to implement evidence-based practices into clinical workflows [4]. PFs receive specialty training to help clinic teams work through complex change processes; they help practices overcome quality improvement barriers, such as fear of change, lack of knowledge, or misperceptions about the value added by implementing a change [5]. Their standardized approaches help address key issues such as establishing clear goals, demonstrating the potential for improvement, providing regular feedback, and trialing changes on a small-scale—all important factors related to securing and maintaining staff motivation and commitment to quality improvement initiatives [6, 7].

Practice facilitation has a strong evidence base for increasing adoption of evidence-based practices and improving care for chronic conditions [1–3, 8]. However, there are a limited number of scales PFs can use to gauge a practice’s progress toward implementing key quality improvement activities and the impact of their work. Of the existing scales, few have psychometric properties established [9]. One measure that PFs may use is called the “Key Driver Implementation Scale” (KDIS). The KDIS items prospectively assess the degree to which a practice implements key quality improvement activities from the Chronic Care Model [10–12]. The KDIS items were developed with stakeholder engagement by experts in quality improvement and practice facilitation for use in primary care [10–12], and are supported by the Agency for Healthcare Research and Quality (AHRQ) [13].

The underlying framework for the KDIS items is the Chronic Care Model [10, 12, 14–17], which assists practices in improving care delivery [18] to strengthen the provider-patient relationship and improve patient outcomes. The KDIS items measure a practice’s progress toward implementing the five key drivers in the Chronic Care Model for a specific quality improvement goal (e.g., improving blood pressure control): a clinical information system; adoption of standardized care processes, optimized team care, use of patient self-management support resources, and practice leadership support [10–12, 19].

The KDIS is used in research and in healthcare quality improvement initiatives across the state. In research, the KDIS has been used in at least 14 randomized trials [20], for an example please see the EvidenceNow trials [21]. Nineteen states have PF programs [22]. One in North Carolina, the North Carolina Area Health Education Center (NC AHEC), routinely uses the KDIS items to assess primary care practices’ progress toward implementing change packages or statewide initiatives. In general, PFs carry a case load of 10–20 primary care practices [23] and engage with practices monthly to assess progress and plan next steps. At each meeting, the PF uses the KDIS to rate the practice on the five key areas described above. The PF’s have access to KDIS responses over time, and review them before or during practice visits as part of continuously strategizing on ways to enhance process and disease outcomes.

Despite this use in clinical trials and state initiatives, the KDIS items have not been psychometrically evaluated. In this study, we examined the psychometric properties of the KDIS items in the Southeastern Collaboration to Improve Blood Pressure Control Study (clinicaltrials.gov #: NCT02866669). This pragmatic, cluster randomized trial compared four arms over one year: (1) practice faciliation, (2) peer coaching, (3) both practice facilitation and peer coaching, or (4) enhanced usual care. The primary outcome was improved blood pressure control for Black adults treated for hypertension in a rural primary care practice. The current study uses the 32 practices randomized to a practice facilitation arm.

PFs completed monthly KDIS ratings for a year at 32 practices. The purpose of the present study was to examine the KDIS items’ psychometric properties including construct validity, reliability, floor and ceiling effects, and a longitudinal trend test. If the KDIS items are found to have low reliability or validity, it may compromise the ability of clinical trials and state initiatives to determine whether the quality improvement goals were met, and thus whether the intervention was effective in improving patient outcomes. Similarly, our construct validity analyses examine whether the KDIS items are measuring one or more groups of quality improvement activities, and thus, provide guidance on whether the KDIS items should be summed or used separately, respectively. Knowing whether to sum the KDIS items or use them separately has implications for assessing factors that may affect PF’s responses as well as outcomes that may be associated with KDIS.

## Methods

### Southeastern collaboration to improve blood pressure control trial

Table 1 shows the details of the Southeastern Collaboration to Improve Blood Pressure Control trial. The trial is pragmatic and cluster-randomized trial with four arms (PF, peer coaching, PF + peer coaching, enhanced usual care) (clinical trials.gov #: NCT02866669). The trial goal is to enhance hypertension control provided by primary care practices serving rural dwelling Black adults with uncontrolled hypertension in North Carolina and Alabama. Patients provided written informed consent with research staff at a participating primary care clinic.

**Table 1:** Parent study synopsis.

**Parent Study Title**	Southeastern Collaboration to Improve Blood Pressure Control Trial
**ClinicalTrials.gov Number**	NCT02866669
**Study Design**	Pragmatic, cluster-randomized trial with 4 arms: 1) 1 year of practice facilitation for primary care practices 2) 1 year of peer coaching for patients enrolled 3) Both 1 and 2 4) Enhanced usual care (Patient Activated Learning System, home blood pressure monitors, practice tips–also provided to arms 1–3 above)
**Number of Primary Care Practices Enrolled**	69
**Number of Practices Randomized to a Practice Facilitator Arm**	32
**Enrollment states**	Alabama and North Carolina
**Number of Practice Facilitators (PFs)**	4 (2 PFs worked with 18 practices in Alabama and 2 PFs worked with 14 practices in North Carolina)
**Primary Trial End Point**	Change in hypertension control by study arm at 12 months
**Primary Care Practice Inclusion Criteria**	• Primary Care practice located in Alabama or North Carolina • Serves a predominately lower socioeconomic and rural population • High proportion of Black patients • Internet access at the practice • Financial stability over study period and no plans to close the practice in the next 3 years • Engagement and commitment by the leadership of the practice to support change • No major disruptions over the study period (e.g., key staff position vacant, new EHR) • Willingness to sign a Letter of Agreement to participate • Willingness to identify a Practice Champion • Willingness to modify structure and processes of care with help of a practice facilitator • Willingness to work with peer coaches

EHR: electronic health record, PFs: practice facilitators

### Practice facilitators

Four PFs (two in Alabama and two in North Carolina) worked with a total of 32 primary care practices between 2017 and 2020 [24]. PFs had a range of 1–5 years of experience working with primary care practices and all had an advanced degree (e.g., Master’s in Business Administration, Public Health Administration). PFs were all certified through the same program at the University of Buffalo, with ongoing training provided by 2 senior PFs from the NC Area Health Education Center practice facilitation team. At the beginning of the study, two practices in Alabama had a PF who left the study early but whom helped train her replacement who remained with the study to its completion. All other PFs remained with the study throughout.

Clinics onboarded at staggered times, which kept the total number of clinics served by any individual PF to 10 or fewer. Each PF worked with the same clinics throughout the study and developed a relationship with each practice. PFs met twice monthly as a group to discuss challenges with their practices and to brainstorm solutions during the active phase of the intervention.

### KDIS items

PFs completed the KDIS items monthly based on their observations and input from the practice, typically while at an in-person visit to the practice. The KDIS has 5 items assessing a practice’s progress toward implementing key quality improvement activities from the Chronic Care Model (see Table 2). The item “clinical information system” assesses the extent to which a practice uses data from their electronic health records or a registry for population health management. “Standardized care processes” assesses use of evidence-based or informed protocols to standardize treatment. “Optimized team care” assesses the extent to which practice team members share workloads for patient care and quality improvement activties. “Patient self-management support” assesses use of resources to enable patients to self-manage their health condition. “Leadership support” assesses a practice’s leadership support for quality improvement activities. Higher scores indicate greater practice involvement in these key activities.

**Table 2:** KDIS items and response options.

KDIS Item	Construct(s) Assessed	Response Options
Clinical Information System	Extent to which a practice uses data from their electronic health record or registry for population health management	0 = Practice currently does not review practice population data, such as a report that shows how many patients have hypertension or how many have it under control.
		1 = Practice has access to reliable data on their patients with hypertension (for example, all patients with hypertension and their average BP, or the % of hypertension patients that have BP <140/90)
		2 = The practice trusts their BP data / reports enough to consider implementing change activities
		3 = The practice accesses and reviews BP data monthly and discusses how to make changes to improve processes to optimize BP control
Optimized Team Care	Extent to which practice team members share workloads for patient care and quality improvement activties	0 = No QI activities related to hypertension currently
		1 = Occasional meetings or discussions regarding QI for hypertension but no practice-wide understanding of QI
		2 = A QI team communicates regularly (through meetings, huddles, emails, memos, etc.) to plan tests and discuss results of hypertension QI. QI team can describe project focus and measures.
		3 = A QI team is planning and discussing multiple tests simultaneously to improve HTN control, and communicates findings to each other. QI progress is communicated to entire office staff. Most staff can describe QI focus and measures.
Standardized Care Processes	Extent to which a practice uses evidence-based or informed protocols to standardize treatment	0 = The practice currently has no activity on following evidence based protocols for hypertension.
		1 = The practice has identified one or more evidence-base or best practice protocol(s) for hypertension, and has begun the process of customizing one or more protocols for their own practice to guide care for their patients with high blood pressure.
		2 = The practice has established a workflow to support implementing at least one hypertension protocol and it has been tested on at least a few patients.
		3 = The practice has implemented an evidence-based protocol for hypertension, but it is not yet being used with all patients.
		4 = The practice routinely fully implements and follows at least one evidence-based protocol for hypertension.
Self-Management Support	Extent to which a practice uses resources to enable patients to self-manage their health condition	0 = Practice currently has no activity on self-management support for patients with hypertension
		1 = Practice staff understands the difference between patient education and self-management support
		2 = Practice identifies hypertension related SMS resources and incorporates the use of the resources into their workflow
		3 = Practice develops tracking systems to monitor use of hypertension related SMS resources.
		4 = The care team 1) collaborates with patients to set hypertension related self-management goals, 2) documents the goals, and 3) reviews previous goals at every visit.
		5 = Care team assess patients’ confidence level related to
		managing their hypertension.
Leadership	Extent to which a practice has leadership support for quality improvement activities	0 = No management or leadership support for QI work in hypertension currently exists.|
		1 = A single manager or physician champion is involved but no organized QI structure for hypertension exists. “Try and see approach” is the norm for QI activities related to hypertension.
		2 = The practice has a leader who supports hypertension QI activities and there are some tasks that are assigned to staff members.
		3 = QI work for hypertension is integrated into daily routines and there are certain staff who are assigned QI activities.

BP: blood pressure, HTN: hypertension, SMS: self-management support, QI: quality improvement

#### Analyses

Table 3 shows an overview of the psychometric analyses. We used a trend test to examine whether the KDIS items increased over time and to estimate the number of months to move a practice to the highest scores. Floor and ceiling effects were assessed with the percentage of practices with the lowest and highest scores in each month, which is important for understanding how sensitive the KDIS items are to changes in practice performance. We assessed construct validity and reliability with a multilevel confirmatory factor analysis. Construct validity examines whether the KDIS items measure one or more groups of distinct implementation activities, which has implications for how KDIS items should be aggregated and interpreted (i.e., whether items should be summed if measuring one dimension or used separately if measuring more than one dimension). Reliability is the degree to which a scale consistently yields the same score. Analyses were conducted using MPLUS 8.0 (Los Angeles, California, USA) or R 4.0.0 (R Foundation for Statistical Computing, Vienna, Austria).

**Table 3:** Overview of psychometric analyses.

Psychometric Characteristic	Statistical Test	What Test Tells Us
Responsiveness	Trend test: Random-intercept linear mixed model with autoregressive residual correlations, treating practice facilitators as clusters	Whether the data shows a statistical trend of increasing scores over time for each KDIS item, and the expected number of months for a practice to move to the highest response options
Floor or Ceiling Effects	Percent of practices in each month scoring zero or the highest response option (low variability in scores)	Floor effects show the percentage of practices scoring consistently at the lowest response option (zero), and ceiling effects indicate the percent of practices scoring consistently at the top of the scale.
Factorial Validity	Multilevel confirmatory factor analysis	Whether the KDIS items measure one or more distinct groups of implementation activities
Reliability	Estimated within the multilevel framework	Whether the scale consistently yields same result

KDIS: Key Driver Implementation Scale

#### Longitudinal trend test

For each KDIS item, a random-intercept linear mixed model with autoregressive residual correlations [25] was fit, treating PFs as clusters. We estimated the fixed effect of time (months 1 through 12) on the KDIS item scores. For better interpretation, we centered the variable for month before fitting the mixed models so that the intercept represents the average item score at month 1.

#### Floor and ceiling effects

We examined the percentage of PF ratings in each month where the lowest response option of zero (floor effect) and highest response (ceiling effect) option was selected. Floor and ceiling effects are one way of identifying where little variance is occurring. There is no gold standard cut-off for a percentage that indicates problematic floor and ceiling effects for practice-level data, although 15–20% is typically used for patient-level data [26]. Thus, we used a 20% cut-off point.

#### Multilevel confirmatory factor analysis and reliability

We first examined the clustering of PFs with intraclass correlation coefficients (ICC) and used 0.01 as a cut-off [27] to determine whether the clustering could be ignored in models. If clustering was significant, we planned to run factor analyses separately by state and use time as a clustering variable. We believe that clustering is more important at the state level than at the PF level (there were two PFs in each state) because state-level policies differ for providing primary care (e.g., Alabama and North Carolina have different Medicaid eligibility criteria, even though neither has expanded Medicaid [28]). Thus, we have provided models separated by state. Essentially, we are ignoring the multilevel/nesting structure under the PF, which typically yields unbiased parameter estimates with biased standard errors [29]. Since we are not interested in the statistical significance of any parameters tested in these models, such biased standard errors would have little impact on our results/conclusions. This dataset is suitable to evaluate the psychometric properties, and is typical of randomized trials and quality improvement inititives where a PF works simultaneously with 10–20 primary care practices to improve care delivery [1, 3, 8].

We then conducted a multilevel confirmatory factor analysis (CFA) for a 1-factor model [29, 30] that combined the 12 months of data. The CFA models used 1 within-level factor and unrestricted covariance at the between level. Given the categorical nature of the KDIS items, the CFA models were fit using a weighted least squares estimator with robust mean and variance adjustments (i.e., the WLSMV estimator), which analyzes polychoric correlations generated for the five items. Model fit was assessed with standard fit criteria [31], including Root Mean Squared Error of Approximation (RMSEA <0.06), Comparative Fit Index (CFI >0.95), Tucker-Lewis Index (TLI >0.95), Weighted Root Mean Square Residual (WRMR <1.0), and Standardized Root Mean Square Residual (SRMR < 0.05) [32]. Reliability was estimated under the multilevel framework [33].

Twelve months of data with 4 PFs working with 32 practices yielded a total of 384 observations. It is not possible to estimate the sample size needed for a multilevel CFA model directly, but a simulation study suggests that a scale with five items (like the KDIS) and a sample size of 32 practices should be acceptable to examine within-level results [34]. Thus, we focused on within-level results for 32 practices rather than the between-level results (by month).

#### Missing values

We conducted tests for missing data that incorporated practice site and month [35, 36]. The assumption of missing-completely-at-random (MCAR) data was not violated under Little’s MCAR test (chi-square = 35.94, df = 28, p = 0.144). The Hawkins test of normality and homoscedasticity [37] also did not show assumption violations (p-value = 0.157). For mixed models, missing data was automatically handled through maximum likelihood. For other analyses not involving mixed modeling, listwise deletion was used.

## Results

**Fig 1:**
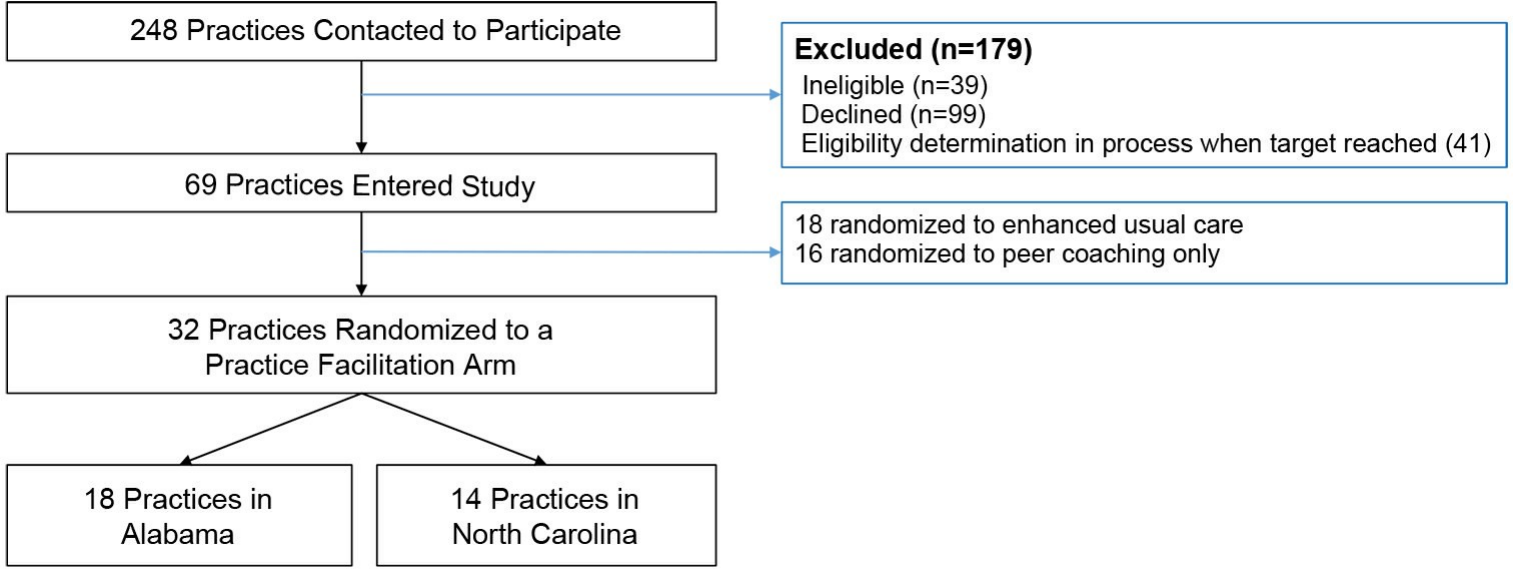
SEC trial CONSORT diagram for primary care practices.

The CONSORT diagram in Fig 1 shows that 69 primary care practices were enrolled and 32 practices were cluster-randomized to a trial arm with practice facilitation.

**Fig 2:**
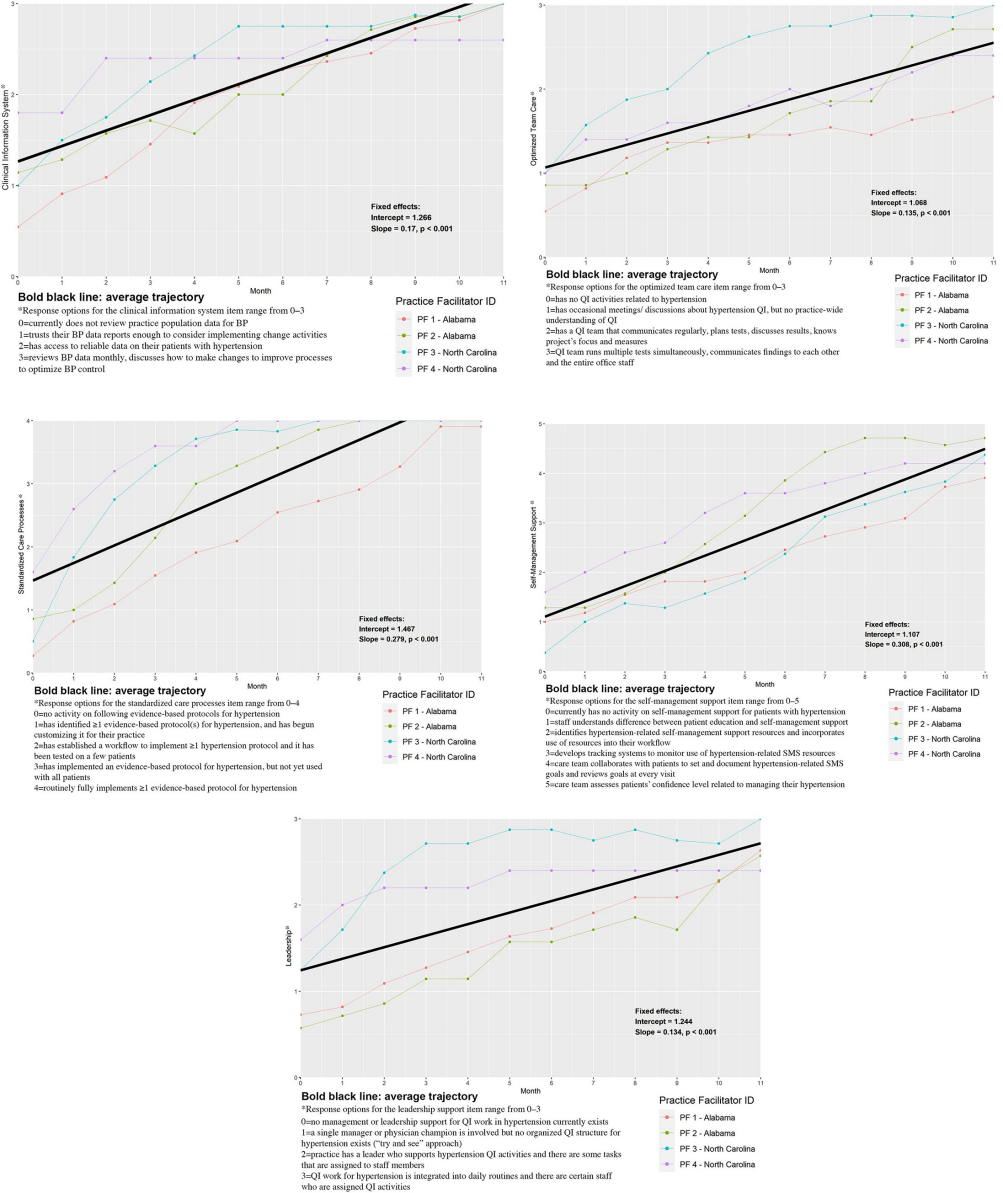
a. Clinical Information System Item Averages for Each Practice Facilitator. b. Optimized Team Care Item Averages for Each Practice Facilitator. c. Standardized Care Processes Item Averages for Each Practice Facilitator. d. Self-Management Support for Patients Item Averages for Each Practice Facilitator. e. Leadership Support Item Averages for Each Practice Facilitator.

Fig 2A–2E are a panel figure where each panel shows the PF trend lines for one KDIS item. In Fig 2A–2E, each color line is the average of one practice facilitator’s ratings for all clinics they worked with over the trial. The dark black line is the average of all 32 practices combined, regardless of PF. PFs 1 and 2 were in Alabama and PFs 3 and 4 were in North Carolina.

Fig 2A–2E show that all KDIS items started at an average of 1 (range: 1.1 to 1.5) on ordinal scales where the lowest response option was zero. KDIS items immediately and consistently increased over time, indicating that practices were increasingly implementing key activities that may influence blood pressure control. Given that all KDIS items started to increase by month 1, it suggests that more global changes may have been occurring. For example, if PFs had waited to start on a specific activity like the clinical information system, the graph would show a flat line at the beginning of the trial until implementation started for that specific task. Instead, Fig 2A–2E show all KDIS items increasing beginning in month 1.

**Fig 3:**
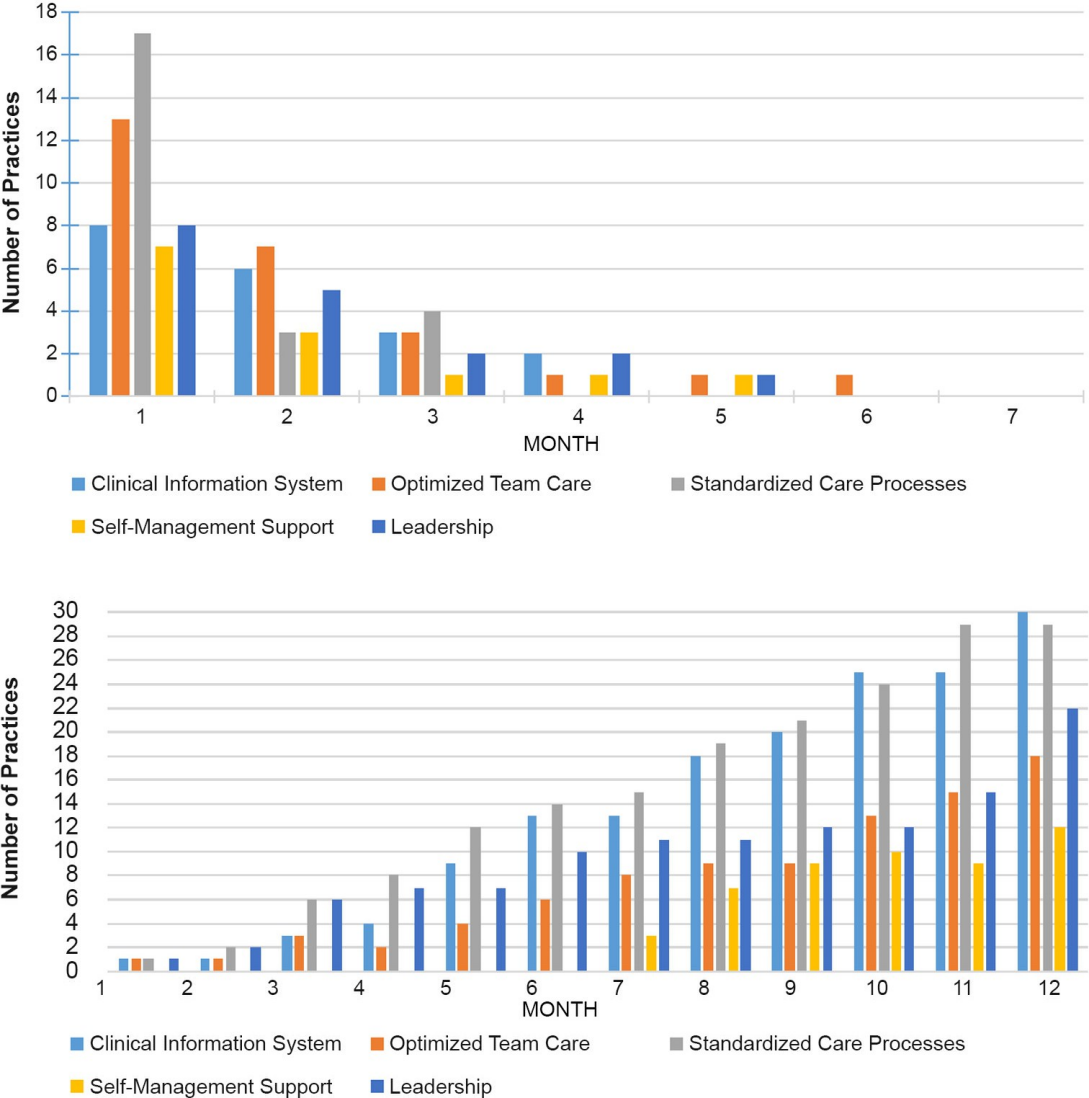
a. Floor Effects by Month. b. Ceiling Effects by Month.

Clustering by state can be seen in Fig 2A–2C and 2E for the KDIS items assessing clinical information system, optimized team care, standardized care processes, and leadership support. The item for patient self-management support does not appear to cluster by state (Fig 2D). Fig 3A and 3B show the floor and ceiling effects by month. In Fig 3A, floor effects (scoring zero) were only significant in month 1. In Fig 3B, ceiling effects (highest response option selected) were significant starting in month 5, indicating low variability in responses in months 5–12.

S1 Table shows the percentage of floor and ceiling effects for each KDIS item by month. We used a cut-off of 20% to show lack of variation [26]. We also looked at whether there was variation in KDIS ratings by PF. S2 Table shows which months had low variation in KDIS ratings for one or more PFs.

Table 4 shows the results of the longitudinal trend test for each KDIS item. Time was used as the fixed effect and PFs were treated as clusters. All KDIS items showed a statistically significant linear trend where scores increased monthly (all t-scores p < .001). Across the 5 KDIS items, the average starting score for practices was 1 (the second highest response option above 0) with a range from 1.068 for optimized team care to 1.467 for standardized care processes. For each KDIS item, the expected increase in score every month ranged from the slowest change of 0.134 and 0.135 for leadership support and optimized team care, respectively, to the quickest change of 0.308 for patient self-management support.

**Table 4:** Longitudinal trend test results for KDIS items.

KDIS Item	Response Options	Intercept: Average Starting Score for Practices	Slope: Expected Increase in Score Every Month	Degrees of Freedom	t-value	Estimated # of Months to Move to Highest Score
Clinical Information System	0 = Practice currently does not review practice population data, such as a report that shows how many patients have hypertension or how many have it under control. 1 = Practice has access to reliable data on their patients with hypertension (for example, all patients with hypertension and their average BP, or the % of hypertension patients that have BP < 140/90) 2 = The practice trusts their BP data / reports enough to consider implementing change activities 3 = The practice accesses and reviews BP data monthly and discusses how to make changes to improve processes to optimize BP control	1.266	0.170	364	18.091***	10.2 months
Optimized Team Care	0 = No QI activities related to hypertension currently 1 = Occasional meetings or discussions regarding QI for hypertension but no practice-wide understanding of QI 2 = A QI team communicates regularly (through meetings, huddles, emails, memos, etc.) to plan tests and discuss results of hypertension QI. QI team can describe project focus and measures. 3 = A QI team is planning and discussing multiple tests simultaneously to improve HTN control, and communicates findings to each other. QI progress is communicated to entire office staff. Most staff can describe QI focus and measures.	1.068	0.135	361	14.589***	14.3 months
Standardized Care Processes	0 = The practice currently has no activity on following evidence based protocols for hypertension. 1 = The practice has identified one or more evidence-base or best practice protocol(s) for hypertension, and has begun the process of customizing one or more protocols for their own practice to guide care for their patients with high blood pressure. 2 = The practice has established a workflow to support implementing at least one hypertension protocol and it has been tested on at least a few patients. 3 = The practice has implemented an evidence-based protocol for hypertension, but it is not yet being used with all patients.|4 = The practice routinely fully implements and follows at least one evidence-based protocol for hypertension. 4 = The practice routinely fully implements and follows at least one evidence-based protocol for hypertension.	1.467	0.279	356	19.95***	9.1 months
Self-Management Support	0 = Practice currently has no activity on self-management support for patients with hypertension 1 = Practice staff understands the difference between patient education and self-management support 2 = Practice identifies hypertension related SMS resources and incorporates the use of the resources into their workflow 3 = Practice develops tracking systems to monitor use of hypertension related SMS resources. 4 = The care team 1) collaborates with patients to set hypertension related self-management goals, 2) documents the goals, and 3) reviews previous goals at every visit. 5 = Care team assess patients’ confidence level related to managing their hypertension.	1.107	0.308	362	25.734***	12.6 months
Leadership	0 = No management or leadership support for QI work in hypertension currently exists.| 1 = A single manager or physician champion is involved but no organized QI structure for hypertension exists. “Try and see approach” is the norm for QI activities related to hypertension. 2 = The practice has a leader who supports hypertension QI activities and there are some tasks that are assigned to staff members. 3 = QI work for hypertension is integrated into daily routines and there are certain staff who are assigned QI activities.	1.244	0.134	363	13.225***	13.1 months

***p < .001

BP: blood pressure, HTN: hypertension, QI: quality improvement

**Fig 4:**
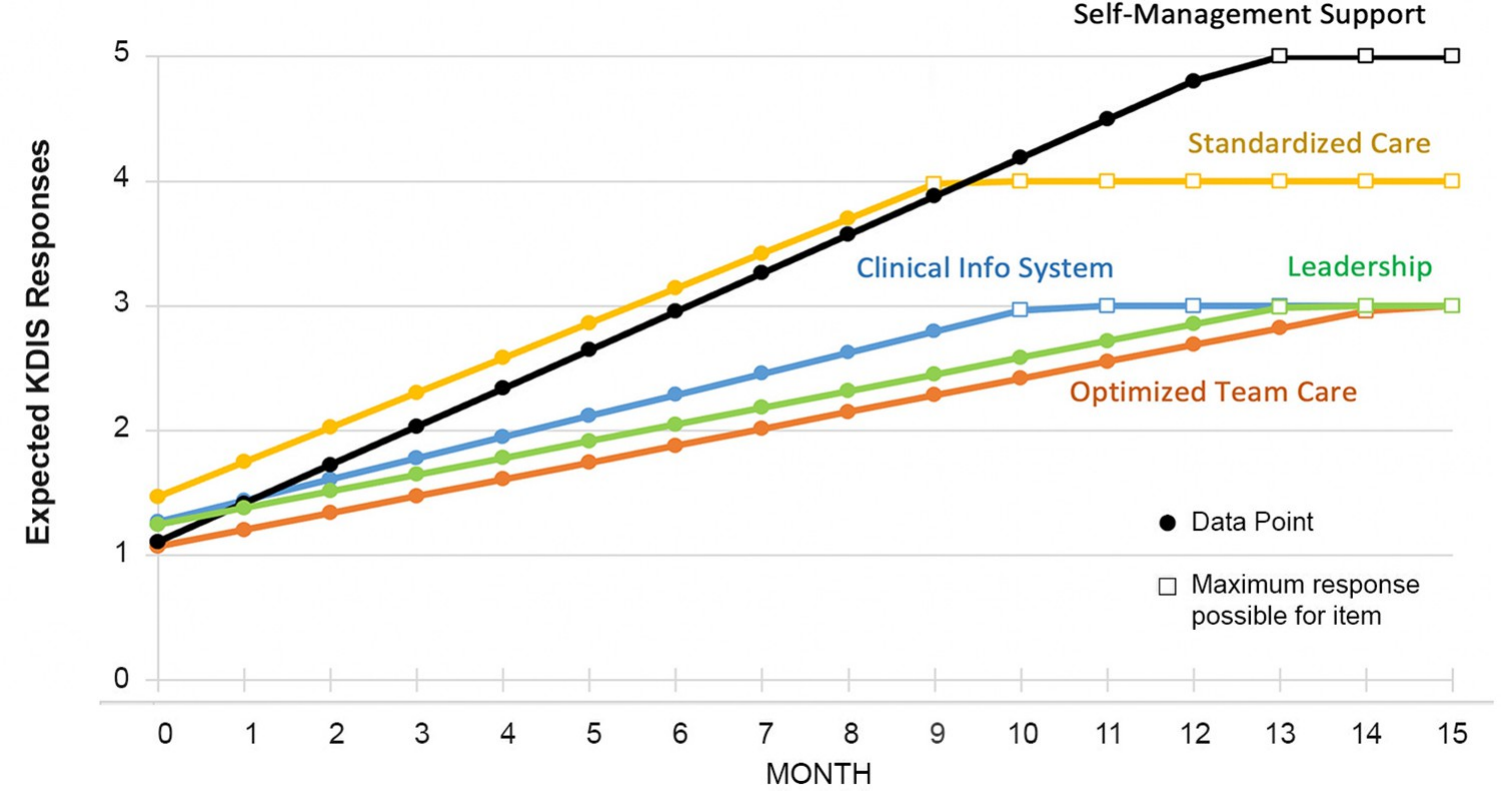
Expected trajectory for KDIS items.

Fig 4 shows the expected trajectory of each KDIS item over 12 months for the 32 primary care practices. All 5 KDIS items showed an expected upward trajectory over time, indicating that practices were increasing their engagement in implementation activities over the course of the study. The expected time to move a practice to the maximum score was 9.1 months for standardized care processes, 10.2 for clinical information system, 12.6 for self-management support, 13.1 for leadership, and 14.3 months for optimized team care.

We initially attempted to fit a multilevel CFA model to the five KDIS items while treating PFs as clusters but the model failed to converge due to estimation errors. As expected, the intraclass correlation coefficients showed that variances due to between-level differences were substantial for all five items (ICCs ranged from 0.352 to 0.787 for Alabama and from 0.245 to 0.757 for North Carolina). Thus, we decided to run CFA separately for practices in Alabama (N = 18 practices) vs. North Carolina (N = 14 practices) and used time (month) as the cluster variable. We had anticipated that clustering at the state level would be important because policies differ by state for providing healthcare (e.g., Alabama and North Carolina have different Medicaid eligibility criteria [28]).

Table 5 shows the multilevel single-factor CFA model results by state, along with the reliabilities of the KDIS scale estimated under the multilevel structure. The composite reliability (omega) was 0.744 for Alabama practices and 0.699 for North Carolina practices, which just meets the minimum threshold of 0.70 for use in group-level analyses. The standardized factor loadings are also inconsistent across states; the most pronounced difference is for patient self-management support where the standardized factor loading is 0.987 for Alabama vs. 0.346 for North Carolina. Given the small sample size and mixed psychometric properties of the KDIS items observed in this trial, the factor structure of KDIS should be examined in larger trials to determine whether the KDIS items should be used separately or as a summed score. These mixed results also suggest that the KDIS item stems and response options may need to be revised to achieve optimal psychometric properties.

**Table 5:** Multilevel confirmatory factor analysis model comparisons by state.

	Alabama	North Carolina
**Within-level reliability**		
Alpha	0.724	0.716
Omega (composite reliability)	0.744	0.699
**Overall model fit**		
Chi-Square Test of Model Fit*	X^2^ = 2.898	X^2^ = 1.724
	df = 5	df = 5
	p = 0.716	p = 0.886
RMSEA <0.06	0.000	0.000
CFI >0.95	1.00	1.00
TLI >0.95	1.192	1.288
WRMR <1.0	0.173	0.068
SRMR within factors <0.08	0.133	0.085
**Standardized factor loadings at the within level**		
Clinical Information System	0.540	0.824
Optimized Team Care	0.705	0.881
Standardized Care Processes	0.812	0.663
Patient self-management support	0.987	0.346
Leadership support	0.601	0.997

* = Test is used to reject a null hypothesis representing perfect fit, and thus the ideal p-value is not significant.

RMSEA = root mean squared error of approximation

CFI = Confirmatory fit index

TLI = Tucker Lewis Index

WRMR = weighted root mean square residual

SRMR = Standardized root mean square residual

## Discussion

Practice facilitation has emerged as an implementation strategy to bridge the gap between research evidence and integrating the evidence in clinical care [1]. PFs may use the “Key Driver Implementation Scale” (KDIS) to measure the degree to which a practice implements key quality improvement activities from the Chronic Care Model: a clinical information system; standardized care processes, optimized team care, patient self-management support to manage their health condition; and leadership support [10, 12]. This is the first study to examine the psychometric properties of the KDIS items.

In the Southeastern Collaboration to Improve Blood Pressure Control Trial, we found that the KDIS items showed mixed psychometric properties. There is room to improve reliability and model fit for the 1-factor confirmatory factor analysis. The standardized factor loadings were unstable between states, and there was marginal reliability. These mixed results suggest that the KDIS items may be measuring more than one group of distinct implementation activities or that the KDIS item stems and response options may need to be revised. A potential multi-factor solution implies that future research should consider using the KDIS items separately, rather than as a sum score. However, we were not able to account for the clustering of the PFs nor conduct exploratory factor analysis due to small sample size. Thus, the factor structure and reliability should be examined in future trials with larger sample sizes.

We also observed ceiling effects starting halfway through the trial (months 5–6). The term “ceiling effect” means different things across fields. We use the term “ceiling effect” to mean low variability in scores, which is a problem because variability is necessary for psychometric and statistical analyses. However, in quality improvement, ceiling effects are viewed positively as a measure of success (practices made it to the desired goal for implementation activities). Below we describe some ways that the KDIS items could be improved to allow for more variability in scores before reaching the highest response options.

We also found that KDIS items showed a significant upward trend over 12 months, suggesting that PFs enabled clinics to advance through implementing key quality improvement activities. In the first month, KDIS items started at an average of 1 (on scales starting at zero) and consistently increased over time, suggesting that PFs were targeting many of the five key drivers at the beginning of work with practices. If PFs had been consistently prioritizing some implementation activities over others at the beginning, we would have observed flat lines for a few months in the areas they were not prioritizing. This consistently upward trend in KDIS items is consistent with other trials using practice facilitation for improving care in Type II diabetes [10, 38] and other chronic conditions treated in primary care [2, 3]. However, a requirement of the trial was at least one quality improvement activity in each of 4 key areas, and thus may not generalize to other trials using practice facilitation.

We also estimated the expected number of months that would be needed on average to move a practice to the highest score for each KDIS item to understand the expected progression and to inform planning of future PF efforts. The expected time to move a practice to the highest response option was 9 months for standardized care processes, 10 for clinical information system, 12.6 for patient self-management support, 13 for leadership support, and 14 months for optimized team care. However, a limitation of the trend test is that we do not know when or how long PFs worked with practices in each area, and which quality improvement activities were primarily PF driven. KDIS responses may reach maximum levels at different rates depending on the focus area(s) targeted by the PF or even the order of activities undertaken. We also do not know exactly how and when practice staff themselves implemented key activities that influenced KDIS scores captured on a monthly basis. KDIS item responses may also have been enhanced due to factors outside the trial (e.g., a practice’s work in population health quality improvement initiatives like the Merit-based Incentive Payment System [MIPS] that are external but complementary to the work in the trial). Such secular trend influences are generally unavoidable in pragmatic trials in real-world practices, and thus it behooves the reader to consider that not all change in KDIS scores may have been directly related to an individual PF’s efforts. This limits our ability to make conclusions about whether certain KDIS score thresholds take longer to achieve than others.

Our estimate of needing up to 14 months to move a practice to the highest response options is consistent with trajectories of practice change for quality improvement initiatives with PFs varying from 5 months [39] to 21 months [40] in the existing literature [1]. From the perspective of PFs, common barriers that take time for them to navigate are team organization and conflicts, challenges with practice engagement (e.g., lack of interest or trust), resistance to change, competing priorities, and using a practice’s electronic health record for quality improvement activities [5, 23]. Ye and colleagues [41] analyzed more than 225 primary care practices receiving practice facilitation in the EvidenceNow trial, and nearly all practices experienced at least one delay toward quality improvement goals during the trial (prior to COVID-19). Practices with more delays had lower intervention completion rates and were more likely to have encountered barriers such as lack of time, staff, and staff engagement, technical issues, and staff turnover [41].

In the current study, the longest interval to reach the highest KDIS response option was expected for optimized team care at 14 months. The KDIS item for optimized team care assesses the extent to which a practice team members share workloads for patient care and quality improvement activties. Reaching the top response category necessitates a practice to not only have a quality improvement team that engages in continuous quality improvement, but also runs multiple quality improvement tests simultaneously, discusses results with the staff, and revises as necessary. Preparing primary care practices to engage in this high level of continuous quality improvement is a complex process that is not well understood [42]. The sustainability of such a continuous quality improvement model when practice facilitation is discontinued is also unknown. Overall, PFs tailored strategies to fit the individual practice needs and helped build data skills and trust in the practice’s own data, but this takes time. Based on our data, future trials using PF could consider increasing the time for PFs to actively work with practices from 12 months to 14 months, when feasible from a trial design and cost perspective.

### Need for increased resources in primary care

PFs are critical for maintaining momentum toward quality improvement goals, but national resources for improving care are in decline. Despite primary care practices being increasingly required to conduct data-driven quality improvement in performance-based payment programs, national resources for building this capacity are dwindling. A recent consensus report [43] highlights that despite primary care providing half of all outpatient visits, it receives a small proportion of resources and research support, has no federal coordinating capacity and a declining workforce pipeline, and remains inaccessible to portions of the population [44]. The consensus report recommends that high-quality primary care be categorized as a common good with public stewardship because of its unique capacity among health care services to improve population health and reduce health care inequities [43]. Importantly, the report also highlights key actions that need to be taken going forward that are consistent with items captured in the KDIS items, including use of interprofessional primary care teams to offset the eroding capacity/maldistribution of primary care clinicians as part of a larger community of care for patients. Within practices, use of effective care team models, such as the Patient Aligned Care Team (PACT) model, have been associated with outcomes such as fewer hospitalizations, fewer specialty visits, less staff burnout, and greater patient satisfaction and other positive outcomes [43].

### Recommendations for future research

Table 6 shows a list of future design considerations for practice facilitation trials and recommendations to improve the psychometric properties of the KIDS items.

**Table 6:** Recommendations and future research agenda.

Recommendations	Rationale
Future Trial Design Considerations	
Add an independent practice rater that evaluates practices on a monthly basis independently of PFs	Examine inter-rater reliability of the KDIS items
Consider increasing the time for PFs to actively work with practices from 12 months to 14 months when feasible in trials	We estimated it may take an expected 14 months for PFs to move practices to the highest response options on some of the KDIS items
Add similar implementation effectiveness scales	Examine convergent and discriminant validity of KDIS items with other scales
Add implementation science outcome variables [45], such as fidelity, adoption, and reach in future PF trials	Increase the robustness of measuring implementation processes and outcomes that can help explain what happened during trial and why
**Future Measurement Work for the KDIS Items**	
Examine the factor structure and reliability in larger sample sizes so the clustering of practice facilitators can be accounted for	Determine whether the KDIS items should be used separately or as a summed score
Examine predictive validity	Examine the extent to which the KDIS items can predict practice and patient outcomes
Develop a KDIS for research use (KDIS-res) that maximizes content validity and psychometric properties	• Content validity can be maximized by developing KDIS-res with input from PFs and primary care practices • KDIS-res may perform better by creating 5 latent constructs with multiple items per latent construct and standardizing response options • Calibrating KDIS-res with item response theory will enable researchers to select the most appropriate items for their trial from a bank of calibrated items [46]

#### Future trial design considerations

The top half of Table 6 is devoted to recommendations for future trial design. For example, we were not able to examine inter-rater reliability for the KDIS items because only one PF rated each practice every month. PFs are professionals who are trained to follow a standardized protocol, but the extent to which individual characteristics influenced their rating is unknown. In the current trial, the four PFs were typical for this professional group in that they were women with an advanced degree and experience in practice facilitation who graduated from the same certificate program. Future research should consider adding an independent practice rater that evaluates practices on a monthly basis independently of PFs to examine inter-rater reliability and whether individual characteristics of PFs influence their KDIS item responses.

We were also not able to examine validity types beyond construct validity (e.g., convergent, divergent, discriminant, and predictive validity). Thus, future work could examine the KDIS items’ predictive validity for practice and patient outcomes. Future PF trials could also add other implementation effectiveness measures to examine convergent and divergent validity and to examine which concepts are unique to the KDIS items. Future trials using PFs would also benefit from adding implementation science measures to further examine the mechanisms of action for implementation [47]. For example, Proctor’s outcome framework [45] or Glasgow and colleagues’ RE-AIM framework [48] could be added to PF trials to assess concepts such as fidelity, adoption, and patient reach that may be missing from existing PF trials.

#### Improving the psychometric properties of the KDIS items

The bottom half of Table 6 includes recommendations to improve psychometric properties of the KDIS items, including developing a research version (“KDIS-res”) that keeps the spirit of the original but improves reliability and the factor structure. Scale development would ideally follow best practices to maximize reliability and validity [46, 49]. NC AHEC is currently updating and expanding the KDIS items they use for healthcare quality improvement initiatives across the state, and this may be a good starting place for creating a KDIS-res.

Content validity of the KDIS-res could be enhanced with ongoing input from PFs and practices via concept elicitation and cognitive interviewing. Currently, each KDIS item has their own unique response set, which are a series of declarative sentences. Each declarative sentence could be developed into its own item and a standardized response set (e.g., “never” to “always”) could be applied across all items. Separating each response option into its own item would lead to each KDIS-res latent variable having multiple items instead of one item like it is currently. For example, the KDIS item assessing “standardized care processes” has four response options that could be separated into at least 4 separate items: 0 = The practice currently has no activity on following evidence based protocols for hypertension; 1 = The practice has identified one or more evidence-based or best practice protocol(s) for hypertension, and has begun the process of customizing one or more protocols for their own practice to guide care for their patients with high blood pressure, 2 = The practice has established a workflow to support implementing at least one hypertension protocol and it has been tested on at least a few patients, 3 = The practice has implemented an evidence-based protocol for hypertension, but it is not yet being used with all patients, and 4 = The practice routinely fully implements and follows at least one evidence-based protocol for hypertension. Response options 1, 2, and 3 are assessing more than one implementation activity (double-barreled) and would likely perform better as separate items. Thus, in a KDIS-res, the subscale for standardized care processes would have a minimum of 7 items.

Conceptually, the content of the KDIS-res items could be enhanced with constructs from implementation science frameworks, such as the “Integrated Promoting Action on Research Implementation in Health Services” (i-PARIHS) [50–52]. i-PARIHS argues that successful implementation of evidence-based practices is based on a PF aligning and integrating the health innovation, recipients, and context. Thus, the KDIS-res items could reflect key constructs in both implementation science and quality improvement. This dual measurement of the overlap between implementation science and quality improvement [47] is already reflected in the ways the KDIS items are used in both routine practice facilitation work by the North Carolina Area Health Education Centers (NC AHEC) [10] and in clinical trials [6, 10, 16, 38, 53].

To maximize the utility of the KDIS-res, the new items would ideally be applicable across care settings, instead of specific to one type of clinic or health condition (the current KDIS items are specific to primary care and hypertension). If an adequate sample size could be achieved, the KDIS-res could be envisioned as an item bank calibrated with item response theory [46]. A calibrated item bank would enable researchers to select items that are fit-for-purpose for the trial being developed instead of the static form used currently.

## Conclusion

In the Southeastern Collaboration to Improve Blood Pressure Control Trial, we found that the KDIS items showed mixed psychometric properties and could be improved. Further psychometric work is needed in larger sample sizes to determine if more than one distinct group of implementation activities is being measured rather than being unidimensional. If two or more factors are shown to be underlying the KDIS items in future research, it would suggest that KDIS items need to be analyzed separately rather than as a total score. The KDIS items also showed low variability and marginal reliability, and thus a research version of the KDIS items (“KDIS-res”) could be developed to improve psychometric properties but keep the spirit of the original items. The longitudinal trend test in this trial suggests that future trials using practice facilitation could consider increasing the number of months of active involvement with primary care practices from 12 months to 14 months, when feasible for trial design and cost.

## Supporting information

S1 Table KDIS floor and ceiling effects (N = 32 practices). (DOCX)

S2 Table Low variation in monthly KDIS rating by practice facilitator. (DOCX)

S3 Table De-identified dataset. (XLSX)

## Abbreviations

BP: blood pressure
CFA: confirmatory factor analysis
CFI: Comparative fit index
EHR: Electronic health record
HTN: Hypertension
KDIS: Key driver implementation scale
PF: practice facilitator
QI: Quality improvement
RMSEA: root mean square error of approximation
SMRM: Standardized root square mean residual
TLI: Tucker-Lewis Index
