# Supplementary material for: The Key Driver Implementation Scale (KDIS) for practice facilitators: Psychometric testing in the “Southeastern collaboration to improve blood pressure control” trial
Source: PLoS One. 2022 Aug 24;17(8):e0272816. doi: 10.1371/journal.pone.0272816 (PMC9401114; doi:10.1371/journal.pone.0272816)
Supplement: S1 Table — (DOCX) [file pone.0272816.s001.docx]

| **Month** | **Clinical Information System (0-3)** | | **Optimized Team Care (0-3)** | | | **Standardized Care Processes (0-4)** | | **Patient Self-Management Support (0-5)** | | **Leadership Support (0-3)** | |
| --- | --- | --- | --- | --- | --- | --- | --- | --- | --- | --- | --- |
|  | **n (%) Floor** | **n (%) Ceiling** | **n (%) Floor** | | **n (%) Ceiling** | **n (%) Floor** | **n (%) Ceiling** | **n (%) Floor** | **n (%) Ceiling** | **n (%) Floor** | **n (%) Ceiling** |
| 1 | 8 (25%)* | 1 (3%) | 13 (41%)* | | 1 (3%) | 17 (53%)* | 1 (3%) | 7 (22%)* | -- | 8 (25%)* | 1 (3%) |
| 2 | 6 (19%) | 1 (3%) | 7 (22%)* | | 1 (3%) | 3 (9%) | 2 (6%) | 3 (9%) | -- | 5 (16%) | 2 (6%) |
| 3 | 3 (9%) | 3 (9%) | 3 (9%) | | 3 (9%) | 4 (13%) | 6 (19%) | 1 (3%) | -- | 2 (6%) | 6 (19%) |
| 4 | 2 (6%) | 4 (13%) | 1 (3%) | | 2 (6%) | -- | 8 (25%)* | 1 (3%) | -- | 2 (6%) | 7 (22%)* |
| 5 | -- | 9 (29%)* | 1 (3%) | | 4 (13%) | -- | 12 (39%)* | 1 (3%) | -- | 2 (7%) | 7 (23%)* |
| 6 | -- | 13 (41%)* | 1 (3%) | | 6 (19%) | -- | 14 (47%)* | 1 (3%) | -- | -- | 10 (32%)* |
| 7 | -- | 13 (41%)* | -- | | 8 (26%)* | -- | 15 (50%)* | 1 (3%) | 3 (9%) | -- | 11 (34%)* |
| 8 | -- | 18 (56%)* | -- | | 9 (28%)* | -- | 19 (61%)* | -- | 7 (22%)* | -- | 11 (34%)* |
| 9 | -- | 20 (63%)* | -- | | 9 (28%)* | -- | 21 (66%)* | -- | 9 (28%)* | -- | 12 (38%)* |
| 10 | -- | 25 (78%)* | -- | | 13 (42%)* | -- | 24 (75%)* | -- | 10 (31%)* | -- | 12 (38%)* |
| 11 | -- | 25 (81%)* | -- | 15 (48%)* | | -- | 29 (97%)* | -- | 9 (30%)* | -- | 15 (48%)* |
| 12 | -- | 30 (94%)* | -- | 18 (58%)* | | -- | 29 (97%)* | -- | 12 (39%)* | -- | 22 (71%)* |

* = Exceeds 20% (problematic lack of variation)
