# Supplementary material for: The Key Driver Implementation Scale (KDIS) for practice facilitators: Psychometric testing in the “Southeastern collaboration to improve blood pressure control” trial
Source: PLoS One. 2022 Aug 24;17(8):e0272816. doi: 10.1371/journal.pone.0272816 (PMC9401114; doi:10.1371/journal.pone.0272816)
Supplement: S2 Table — (DOCX) [file pone.0272816.s002.docx]

| **KDIS Item** | **No Variation in KDIS Rating** | | | | | | | | | | | |
| --- | --- | --- | --- | --- | --- | --- | --- | --- | --- | --- | --- | --- |
|  | **Month** | | | | | | | | | | | |
|  | **1** | **2** | **3** | **4** | **5** | **6** | **7** | **8** | **9** | **10** | **11** | **12** |
| **Clinical Information System**  0-3 scale |  |  |  |  |  | PF 2  (rated all as 2) | PF 2  (rated all as 2) |  |  |  |  | PF 3  PF 2  PF 1  (rated all as 3) |
| **Optimized Team Care**  0-3 scale |  |  |  |  |  |  |  |  |  |  |  | PF 3  (rated all as 3) |
| **Standardized Care Processes**  0-4 scale |  |  |  |  |  | PF 4  (rated all as 4) | PF 4  (rated all as 4) | PF 4  PF 3  (rated all as 4) | PF 4  PF 3  PF 2  (rated all as 4) | PF 4  PF 3  PF 2  (rated all as 4) | PF 4  PF 3  PF 2  (rated all as 4) | PF 4  PF 3  PF 2  (rated all as 4) |
| **Self-management support for patients**  0-5 scale | PF 1  (rated all as 1) |  |  |  |  |  |  |  |  |  |  |  |
| **Leadership**  0-3 scale |  |  |  |  |  |  |  |  |  |  |  | PF 3  (rated all as 3) |
